# Effect of Gaseous Ozone Process on Cantaloupe Melon Peel: Assessment of Quality and Antilisterial Indicators

**DOI:** 10.3390/foods10040727

**Published:** 2021-03-30

**Authors:** Fátima A. Miller, Joana F. Fundo, Ester Garcia, Cristina L. M. Silva, Teresa R. S. Brandão

**Affiliations:** Laboratório Associado, Centro de Biotecnologia e Química Fina, Universidade Católica Portuguesa, CBQF, Escola Superior de Biotecnologia, Rua Diogo Botelho 1327, 4169-005 Porto, Portugal; fmiller@porto.ucp.pt (F.A.M.); jfundo@porto.ucp.pt (J.F.F.); esther_egr3@hotmail.com (E.G.); clsilva@porto.ucp.pt (C.L.M.S.)

**Keywords:** melon peel, quality/safety, ozone, bioactive compounds, *Listeria* spp.

## Abstract

Fruit waste parts, particularly peel, are abundant sources of bioactive compounds. To be included in the formulation of value-added foods, peel needs to be transformed and subjected to a preservation process. Therefore, this study seeks to assess the effect of ozone on the quality and antilisterial indicators of cantaloupe melon peel paste, aiming at obtaining a product with the potential to be used as a food additive. Ozone was bubbled during 30 and 60 min, and some physicochemical characteristics (soluble solids content, pH and colour), bioactive compounds (total phenolics, chlorophylls and vitamin C) and antioxidant activity were analysed. Peel was also inoculated with *Listeria innocua*, used as a treatment efficiency indicator. The results indicated that, although ozone negatively affected antioxidant activity, it positively influenced all bioactive compounds analysed. An *L. innocua* reduction of 1.2 log cycle was achieved after ozone exposure. Ozone should be exploited as a promising technology to assure the quality/safety of cantaloupe melon peel. Indeed, if melon peel is conveniently converted into a suitable form that can be used as a food ingredient, this will promote the valorisation of waste materials with the consequent reduction of industrial by-products and new perspectives for market opportunities.

## 1. Introduction

Melon fruit is consumed fresh or processed in the form of juices, nectars or jams. During industrial processing, high quantities of peel are produced, representing a severe problem by negatively impacting the environment. However, these wastes are an abundant source of bioactive compounds and nutrients, known to have a favourable effect on health [1].

Besides exploiting the peel nutritive profile, this waste part’s transformation aims to obtain a highly nutritious product in an appealing and convenient form. The transformed product shall be a source of bioactive and nutritional compounds that can be used (e.g., pastes) to formulate foods. Some studies reported applications of the peel of several fruits as food ingredients. Kanatt et al. [2] studied the effect of pomegranate peel addition on chicken and meat products, obtaining a shelf-life increase of two to three weeks. Additionally, an effective control of oxidative rancidity was attained in these products when the peel extract was incorporated. de Moraes Crizel et al. [3] used orange peel fibre to reduce the fat content of ice cream by 70% without significantly affecting quality parameters such as the colour, texture and aroma. Ajila et al. [4] reported a potential use of mango peel as an ingredient of bakery products, breakfast cereals, bars and beverages.

Fundo et al. [5] assessed Cantaloupe melon peel’s potential as a source of health-related compounds such as potassium, chlorophylls, total phenolics and antioxidant activity. However, and as far as our knowledge is concerned, the transformation of melon peel into suitable forms that can be applied as food ingredients is not yet exploited. One possible way is to transform the peel into a paste by using industrial homogenisers incorporated into several food products like smoothies, nutritional bars and snacks, yoghurts, or bakery items, enhancing the nutritional and bioactive profile of these products. Peel transformation into paste requires the application of preservation processes for a shelf-life extension. However, such methods may negatively affect the nutritive product profile. Therefore, a careful assessment of the impact of a given process on biocomponents is crucial, by seeking the development of new strategies for adding value to wasted materials.

It is well known that heat treatments significantly affect health-related compounds. Therefore, nonthermal technologies are being exploited. Ozone is an environmentally friendly processing that has been extensively used over the years due to its efficiency in water and food treatments [6,7].

Research on the influence of ozone in fruit peel is scarce. However, several studies reported gaseous ozone’s effectiveness on the quality and safety of Cantaloupe melon. Selma et al. [8] investigated the effect of a gaseous ozone application in cantaloupe melons on their microbial and sensory quality. They observed a decrease in the initial counts of mesophilic and psychotropic bacteria, moulds and coliforms when ozone was applied, and no impact on quality attributes. Another study developed by the same authors, but this time in fresh-cut cantaloupe melon, also revealed that ozone exposure significantly reduced all microbial counts and that quality attributes were maintained [9].

Melon peel is very susceptible to contamination since the fruit grows over the soil. *Listeria monocytogenes* is one of the microorganisms of significant concern due to its ubiquitous characteristics and pathogenicity. It has been isolated from several habitats such as soil and vegetation and due to its features may survive in minimally processed foods, which is the case for ozone-treated ones. Because of their similarities and the fact that *Listeria innocua* is a nonpathogenic species, it is often chosen instead of *L. monocytogenes* [10].

This work’s main goal was to assess the effect of gaseous ozone treatments, applied as a preservation process, on the physicochemical and nutritional profile of cantaloupe melon peel paste. The treatment’s impact on *L. innocua* inactivation was also investigated.

## 2. Materials and Methods

### 2.1. Samples

‘Cantaloupe’ melons (*Cucumis melo* L. var. *reticulatus*) were acquired in a local store. The peel was removed from the fruit, cut into small pieces using a knife and triturated with a domestic blender to achieve a homogenised paste, as explained in Fundo et al. [5].

### 2.2. Ozone Treatment

**Figure 1:**
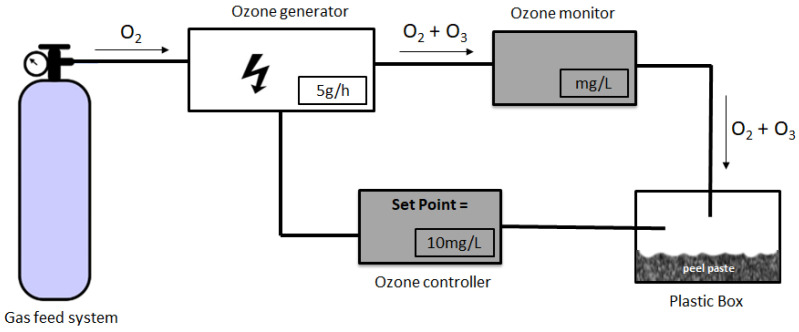
Experimental setup of the ozonation system used.

Ozone (O_3_) treatments were carried out in a pilot plant (Figure 1). The system includes an oxygen tank (with pure oxygen) interconnected to an ozone corona discharge generator (OZ5, SPO3—Sociedade Portuguesa de Ozono, Lda, Porto, Portugal).

The ozonator capacity was 5 g/h, and the gaseous ozone produced was continuously ejected by teflon^®^ (polytetrafluoroethylene) piping (internal diameter of 6 mm) into a hole in a hermetic plastic box (12 × 27 × 17 cm) with a thick film (≈0.5 cm) of 50 g of peel paste at 4 °C. The ozone concentration in the gas stream was monitored continuously and determined, as described by Miller et al. [11]. The system included an ozone controller with a set-point of 10 mg/L. The experiments were initiated when this value was attained (c.a. 3 min to reach equilibrium). For ozone exposure times of 30 and 60 min at a controlled temperature of 15 °C, the corresponding ozone doses were 152 ± 71 and 369 ± 193 ppm, respectively. At the end of treatments, the ozone generator was turned off and the sample containers (plastic box in Figure 1) were opened for aeration when the controller did not detect the presence of ozone.

Each treatment condition was repeated three times.

### 2.3. Evaluation of Physicochemical Properties

#### 2.3.1. Soluble Solids Content and pH

The soluble solids content (SSC, °Brix) and pH of the homogenised peel were measured for each treatment after filtration with cheesecloth. SSC was assessed with a Palette PR-32 digital refractometer (Atago, Tokyo, Japan). A pH meter (GLP 22, Crison Instruments, Barcelona, Spain) was used to quantify the pH values.

The titratable acidity (TA) was also evaluated to ensure the calculation of the melon initial maturity state. Titration was conducted with a 0.1 M NaOH (Pronalab, Lisbon, Portugal) to pH 8.1, and TA was expressed as mmolH^+^/L of the sample. For the maturity index determination, the following equation was used:

(1) $$\mathrm{Maturity}\;\mathrm{index}\;\left(\mathrm{MI}\%\right)=\frac{\mathrm{Soluble}\;\mathrm{solids}\;\mathrm{content}}{\mathrm{Titratable}\;\mathrm{acidity}}\times 100$$

#### 2.3.2. Colour Properties

The peel pastes’ colour was measured using a Minolta CR-400 colourimeter (Konica-Minolta, Osaka, Japan). The samples were analysed in duplicate, and three readings were performed. From the CIELAB colour components (L* a* b*), several colour parameters were calculated as follows:

(2) $$TCD=\;\sqrt{{\left(L_{0}^{*}-L^{*}\right)}^{2}+{\left(a_{0}^{*}-a^{*}\right)}^{2}+{\left(b_{0}^{*}-b^{*}\right)}^{2}}$$

where the index “0” is related to a fresh sample.

(3) $$Chroma=\;\sqrt{{a^{*}}^{2}+{b^{*}}^{2}}$$

(4) $$Hue\;angle=tan^{-1}\left(\frac{b^{*}}{a^{*}}\right)$$

The TCD (Total Colour Difference reflects the colour change of processed samples in comparison to the fresh sample. The saturation index or chroma and the hue angle provide more information about the spatial distribution of colours than the direct values of tristimulus measurements [12].

### 2.4. Bioactive Compounds Analysis

#### 2.4.1. Total Phenolics

The phenolics contents were determined using the Folin–Ciocalteu method [5], by blending 25.0 g of peel samples with 50.0 mL of 100% methanol (Merck, Darmstadt, Germany) and using an ultra-turrax homogeniser (Ika digital T25, IKA-Werke GmbH & Co. KG, Staufen, Germany). The results were expressed as μg gallic acid per g of melon peel.

#### 2.4.2. Vitamin C Determination

Vitamin C (ascorbic acid (AA) plus dehydroascorbic acid (DAA)) in melon peel was quantified by a high-performance liquid chromatography (HPLC) analysis, with a reverse phase C18-silica analytical column (Waters Spherisorb ODS2 5 µm 4.6 × 250 mm) using the method described by Zapata and Dufour [13]. The mobile phase, standard solutions and samples were prepared as explained by Fundo et al. [5].

The results were expressed in mg of vitamin C per 100 g of melon peel.

#### 2.4.3. Chlorophylls Determination

Chlorophylls *a* and *b* were extracted by blending 7.0 g of melon peel with 50.0 mL of 100% methanol with an ultra-turrax. The extracts were centrifuged at 5000× *g* at 4 °C for 10 min and posterior filtered with filter paper. Chlorophylls *a* and *b* quantification (expressed in µg per gram of peel) was determined spectrophotometrically and calculated using Equations (4) and (5) (µg/mL) [14].

(5) $$\mathrm{Chlorophyll}\;a=16.72\;A_{665.2nm}-9.16\;A_{652.4nm}$$

(6) $$\mathrm{Chlorophyll}\;b=34.09\;A_{652.4nm}-15.28\;A_{665.2nm}$$

The total chlorophyll content was calculated from the sum of chlorophyll *a* and chlorophyll *b*.

### 2.5. Total Antioxidant Activity

The ABTS assay was used to determine the total antioxidant activity according to Fundo et al. [5], with the results being expressed as μg of AA per gram of melon peel.

### 2.6. Microbiological Analysis

#### 2.6.1. Culture

*Listeria innocua* 2030c, obtained by the Public Health Laboratory Service—PHLS (Colindale, UK) private collection, was subcultured as described by Miller et al. [15] in order to attain an initial concentration of approximately 7 log cfu/mL. This *L. innocua* concentration in suspension was always used for inoculation.

#### 2.6.2. Peel Inoculation and Microbial Enumeration

Treatments with ozone were performed as previously described using artificially inoculated peel paste. Approximately 35.0 g of sample was immersed during 1 min in 100 mL of bacterial suspension, and excess was discarded. For enumeration, 225 mL of sterile peptone water (BPW, Merck, Darmstadt, Germany) was added to approximately 25.0 g of peel paste into stomacher bags and pummelled for 2 min at normal speed. *L. innocua* was determined following the spread plate method on Palcam agar (Merck, Darmstadt, Germany) at an incubation temperature of 37 °C for 48 h. Microbial counts were achieved in duplicate and expressed as cfu/mL.

### 2.7. Statistical Analysis

A one-way ANOVA was used to detect significant differences between fresh and ozone-processed peel paste for 30 and 60 min concerning all of the analysed characteristics. Tukey’s test was posteriorly performed to assess pairwise differences of means. The normality and homoscedasticity of the data were assessed using Shapiro-Wilk and Levene’s tests, respectively.

When the normality of data was not verified, the nonparametric Kurskal-Wallis test was carried out alternatively to a one-way ANOVA with a Mann–Whitney test for the further detection of differences. The significance level was set at 5% in all cases. The results were expressed as mean ± margin of confidence interval at 95%. All analyses were performed using IBM SPSS Statistics 24 for Windows^®^ (SPSS Inc., Chicago, IL, USA).

## 3. Results and Discussion

### 3.1. Effect of Ozone Exposure on Physicochemical Parameters

The influence of ozone treatments on the pH and SSC can be seen in Table 1. No significant differences in SSC values were observed between fresh and ozone-treated samples. These results are in agreement with the majority of published researches [16], as well as the ones reported using fresh-cut Cantaloupe melon [8,9]. Although 30 min of gaseous ozone exposure did not significantly affect the pH value, 1 h of treatment significantly reduced this parameter. This means that treatment time is relevant for pH maintenance.

The maturity index parameter is the equilibrium between the SSC (balance of sugars and acids present in a matrix) and TA. It is a useful way to determine the internal fruit maturation [17]. The value obtained for this parameter was 80.57, which is in the lower range limit reported in the literature [18,19,20].

The influence of ozone treatments on the colour parameters L* (brightness), a* (greenness) and b* (yellowness) achieved for peel paste samples are also presented in Table 1, as well as the calculated TCD, chroma and hue angle. No significant differences were detected for all colour parameters. For a global perception of the changes that occurred in the colour between the untreated and treated samples, the TCD parameter was calculated. According to Drlange [21], the total differences obtained are considered between ‘very distinct’ and ‘great’, since the values were higher than 3 and lower than 12. However, the treatment time did not significantly affect this parameter. Concerning the chroma parameter, the values were all in the same magnitude, which means that fresh and ozone-treated peel paste presented the same colour intensity. The hue angle defines the colour (red is placed at 0°, yellow at 90°, green at 180° and blue at 270°) [22]. As can be observed, this colour parameter was situated between yellow and green for all untreated and treated samples, which was related to the presence of chlorophylls. Generally, the literature reports no fruit colour changes after ozone treatments [16]. This result was also detected by Selma et al. [9] in cantaloupe fresh-cut melon cubes, after gaseous ozone treatments at 5000 and 20,000 ppm.

### 3.2. Effect of Ozone Exposure on Bioactive Compounds

**Figure 2:**
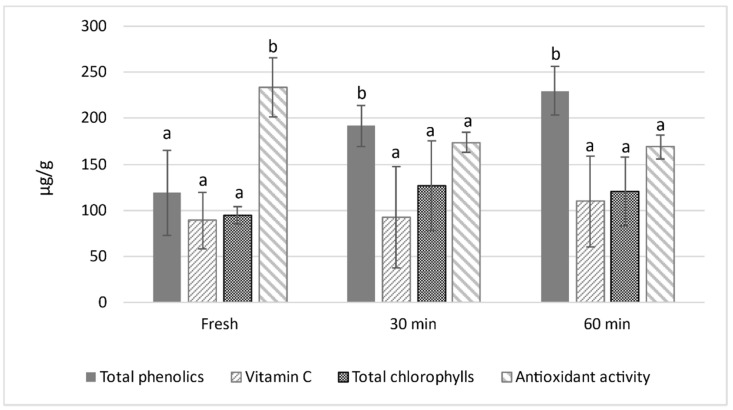
Total phenolics, chlorophylls, vitamin C and antioxidant activity in fresh and ozone-treated cantaloupe peel paste. Data are mean values, and the limits of the bars are confidence intervals at 95%. For a given characteristic, values with the same letters do not differ significantly (*p* > 0.05).

The influence of ozone treatments on the bioactive compounds of melon peel samples was performed in terms of total phenolics, vitamin C and total chlorophylls. These results are displayed in Figure 2.

As can be observed, ozone treatments significantly increased the total phenolics content after 30 min of exposure. This increase may be the result of fruit defence mechanisms induced by the oxidative stress caused by ozone intense oxidising activity [16,23]. The accumulation of phenolic compounds, possibly by the activation of pre-existing enzymes such as phenylalanine ammonia-lyase (PAL), a key regulatory enzyme in the biosynthesis of phenolic compounds, was reported to be induced after ozone exposure [24]. Booker and Miller [25] also observed an increase in caffeic and p-coumaric acids of soybean leaves due to the induction of PAL after ozone treatments. However, according to Sachadyn–Król et al. [26], the increase in the concentration of the total phenolics content in the ozone-treated fruit products can also result from transport rather than enhanced synthesis. In their study, prolonged ozone exposure induced a strong system reaction involving the transportation of antioxidants from internal tissues to the site of oxidative stress.

As shown in Figure 2, ozone exposure slightly increased the total vitamin C content on melon peel samples, especially after 60 min of treatment time. However, this variation was not significantly different. Vitamin C, also named ascorbic acid (AA), also includes its oxidised form, dehydroascorbic acid (DHA). AA is one of the most abundant and essential antioxidants present in intact plant cells [23]. However, much higher DHA values (86.82 ± 29.26 mg/g) were observed for the fresh peel when compared with AA (2.15 ± 1.17). This may be a consequence of cell rupture during peel paste preparation, and the subsequent degradation of AA into DHA.

Chlorophylls are an essential quality parameter, since they display several biological properties, such as antioxidant and antimutagenic activities [27]. The contents of chlorophylls *a* and *b* were determined since they are the primary forms found in fruit peel [28,29]. Although the content in chlorophylls *a* and *b* increased right after 30 min of ozone exposure, this increase was not significant enough to affect the total chlorophylls’ content (Figure 2). After ozone exposure, this slight increase is probably due to the inhibition of chlorophyll-degrading enzymes or the induction of antioxidants that can protect chlorophylls [23].

### 3.3. Effect of Ozone Exposure on Total Antioxidant Activity

The presences of phenolics, vitamin C, chlorophylls, anthocyanins and carotenoids are the main factor that influences fruits’ antioxidant capacity. As shown in Figure 2, antioxidant activity significantly decreased after 30 min of treatment (25%) due to ozone intense oxidising action [16]. However, the treatment time did not statistically affect this parameter.

Since all antioxidant compounds analysed in this study (vitamin C, total chlorophylls and total phenolics) increased after ozone exposure, the detected reduction in the total antioxidant activity could be linked with the decrease of other antioxidants, such as enzymes.

**Figure 3:**
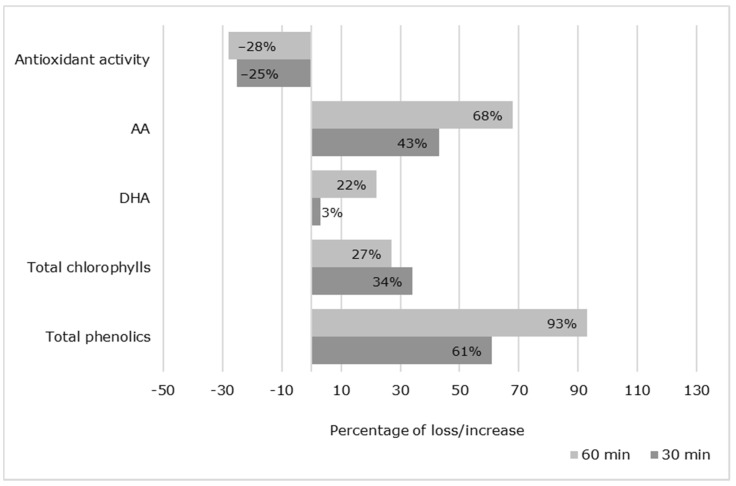
Bioactive compounds and antioxidant activity changes (in percentages) after ozone treatments. AA: ascorbic acid; DHA: dehydroascorbic acid.

For a better awareness of the antioxidant variations resulting from ozone exposure, Figure 3 presents their loss/increase in percentages compared to untreated samples.

All antioxidant compounds increased, except for the total antioxidant activity. The parameter that presented the highest increase was total phenolics, with an increase of almost 100% after 60 min of ozone exposure. AA was also profoundly affected, with an increase of around 50%. This result may be due to the activation of an antioxidative system in response to ozone exposure, promoting the biosynthesis of vitamin C from plant carbohydrate reserves [30]. Nevertheless, DHA was the bioactive compound that was shown to be less sensitive to ozone exposure, with only a 3% and 22% increase after 30 and 60 min of treatment, respectively.

### 3.4. Effect of Ozone Exposure on Listeria Innocua Inactivation

The reduction in the *L. innocua* level on cantaloupe peel paste, at a gaseous ozone concentration of 31.0 g/L, was determined after 30 and 60 min of treatment. The initial level of *L. innocua* in peel paste, determined as 5.8 log cfu/mL, decreased by 1.23 ± 0.10 log after 30 min and 1.24 ± 0.28 log after 60 min of ozone exposure. The treatment time did not affect *L. innocua* inactivation, since after 60 min the microbial level remained the same. This microbicidal ozone action can be related to the damage of several cellular constituents, including unsaturated lipids, proteins and respiratory enzymes in cell membranes, enzymes and nucleic acids in the cytoplasm, and proteins and peptidoglycan in spore coats and virus capsids [6,16].

The effectiveness of ozone treatments has been investigated for many bacteria and food matrices. Selma et al. [9] studied the influence of gaseous ozone in decreasing a population of *Salmonella poona* inoculated on cantaloupe rind. After 30 min of ozone exposure at 10,000 ppm, they obtained a minimum reduction of 1.4 log cfu/mL. Trinetta et al. [31] also explored the effect of gaseous ozone on cantaloupe seeds and concluded that this treatment (4.3 ppm for 5 min) resulted in a decrease of 4.29 log cfu/g of *S. poona*. Another study, developed by Rodgers et al. [32], used cantaloupe melon to evaluate the usefulness of aqueous ozone (3 ppm for 5 min) for *Escherichia coli* and *L. monocytogenes* inactivation. They concluded that ozone exposure reduced the microorganisms’ population by 5.6 logs.

The antimicrobial efficacy of ozone on microbial load depends on numerous issues, which explains the diversity of published results. Some of the main influential factors include the type of food product, target bacteria, initial microbial load, physiological state of the bacterial cells and physical ozone state [16].

## 4. Conclusions

Ozone treatments did not significantly affect any physicochemical parameters that were analysed on melon peel paste, except for pH. Regarding bioactive compounds, ozone had a positive effect by promoting an increase of vitamin C, total phenolics, and chlorophylls after 30 min of exposure. The total phenolics were the most affected parameter, with an increase of almost 100% after 60 min of ozone treatment.

Additionally, ozone treatment was also able to inactivate 1.23 ± 0.10 log cfu/mL of *L. innocua* after 30 min of treatment, with no differences between the treatment times.

Since ozone did not have a negative effect on the quality parameters, it should be combined with other nonthermal technologies, such as ultrasounds and ultraviolet-C radiation, in order to improve the antimicrobial effectiveness. This work also allowed us to conclude that cantaloupe peel is an essential nutritional source that retains its main characteristics even after applying a powerful oxidising agent, and that it will therefore be exploited.

## Figures and Tables

**Table 1:** Colour parameters, pH and SSC of fresh and ozone-treated peel paste. The values are mean ± margin of confidence interval at 95% ^†^.

Peel	Colour Parameters						pH	SSC (°Brix)
	L*	a*	b*	TCD	Chroma	Hue Angle (°)		
Fresh	45.46 ± 5.37 ^a^	−12.70 ± 0.45 ^a^	31.71 ± 2.57 ^a^	-	34.19 ± 2.38 ^a^	111.99 ± 1.88 ^a^	5.90 ± 0.23 ^b^	6.21 ± 0.38 ^a^
30 min O_3_	39.15 ± 6.29 ^a^	−12.71 ± 0.71 ^a^	28.66 ± 4.94 ^a^	8.79 ± 3.65 ^a^	31.43 ± 4.68 ^a^	114.58 ± 3.31 ^a^	5.64 ± 0.13 ^a,b^	6.67 ± 0.31 ^a^
60 min O_3_	41.91 ± 4.60 ^a^	−12.57 ± 0.77 ^a^	29.53 ± 3.42 ^a^	5.46 ± 3.21 ^a^	32.15 ± 3.17 ^a^	113.39 ± 2.76 ^a^	5.10 ± 0.60 ^a^	6.48 ± 0.29 ^a^

^†^ Margin of 95% confidence interval = (confidence intervals at 95%)/2. For a specific characteristic, values with the same letters are not significantly different (*p* > 0.05).

## Data Availability

The datasets generated for this study are available on request to the corresponding author.
